# Functional network comparative area and topography analysis (FUNCATA) in non-affective psychosis: a replication study

**DOI:** 10.1038/s41537-026-00736-z

**Published:** 2026-02-17

**Authors:** Daniel Mamah, ShingShiun Chen, Michael P. Harms, Mark Curtis, Fanghong Dong

**Affiliations:** 1 Department of Psychiatry, Washington University School of Medicine, St. Louis, MO USA; 2 Department of Psychological & Brain Sciences, Washington University, St. Louis, MO USA

**Keywords:** Psychosis, Biomarkers

## Abstract

Resting-state fMRI studies consistently demonstrate widespread dysconnectivity in schizophrenia (SZ), yet conventional analytic methods often fail to account for individual variability in functional brain organization. This study utilized individualized assessments of network size and topography to examine functional alterations in early psychosis. MRI data were drawn from the Human Connectome Project – Early Psychosis study (ages 18–34), including 86 individuals with non-affective psychosis (NAP) and 57 healthy controls (HC). Ten large-scale functional networks were delineated using template-matching procedures. Group differences in network size were evaluated using ANOVA, while network topography was examined with vertex-wise chi-square analyses and the Topographic Abnormality Index (TAI). Compared to controls, NAP participants showed significantly larger dorsal attention (DAN) and default mode (DMN) networks, along with a smaller sensorimotor-body (SBN) network (effect sizes d = 0.39–0.48). NAP also exhibited greater topographic abnormalities in the DAN, DMN, and cingulo-opercular (CON) networks. DMN size was inversely related to mania symptoms, antipsychotic treatment duration, and working memory performance, while smaller SBN size was also linked to reduced working memory. A k-means clustering revealed three psychosis biotypes. Biotype 1 had enlarged DAN and language network size, with higher antipsychotic exposure. Biotype 2 showed near-normal network profiles but elevated mood symptoms. Biotype 3 exhibited enlarged DMN/DAN and reduced frontoparietal network size, with prominent negative symptoms. Consistent with prior schizophrenia studies, DAN enlargement was present in early psychosis, suggesting stability across illness stages. Altered DAN and DMN organization may serve as early biomarkers to guide detection and intervention strategies.

## Introduction

Despite advancements in research and treatment, schizophrenia remains a major public health challenge, with significant personal, societal, and economic burdens^1^. Although prior studies have identified changes in brain structure, connectivity, neurotransmitter systems, and inflammation, the precise mechanisms driving the disorder remain unclear^2–5^. Advancing our understanding of schizophrenia’s pathophysiology is key to identifying novel therapeutic targets and developing precision medicine approaches that improve symptom management and long-term recovery^6,7^.

Resting-state functional magnetic resonance imaging (rs-fMRI) offers a powerful tool for identifying brain-based biomarkers by measuring spontaneous low-frequency BOLD signal fluctuations at rest, which reveal functional connectivity (FC) patterns^8,9^. Many studies using rs-fMRI in schizophrenia have produced varied findings^10–14^, though a consistent pattern is widespread hypoconnectivity, suggesting disrupted functional networks^10,13,14^.

A major challenge in connectivity studies is the high variability in functional network topography across individuals^15,16^. Traditional analyses often use group-average parcellations to assess connectivity strength^17–21^, but these predefined maps may not align with true functional regions in individual brains due to variability in organization^15,22–27^. This can introduce unwanted variance and mislead interpretations, especially when regions of interest (ROIs) do not match functional boundaries. In such cases, findings may reflect noise rather than true network differences^28^. Importantly, variability in network size and topographic alignment itself can systematically bias estimates of FC strength. For example, when functional boundaries are misaligned, connectivity may appear reduced simply because portions of the true network are omitted or combined with unrelated tissue. Conversely, apparent hyperconnectivity can emerge when enlarged or displaced networks artificially increase shared signal. Thus, alterations in network size and spatial configuration—features increasingly recognized in schizophrenia—may directly shape observed FC abnormalities and contribute to the mixed findings reported in the literature^10–14^. To address this, more advanced methods—such as individualized parcellation or data-driven techniques—are needed to account for this heterogeneity. Improving ROI precision is essential to enhance reliability and interpretability of connectivity research, ultimately leading to better insights into brain disorders^15,22–28^.

Functional network size and topographic organization are increasingly recognized as meaningful neurobiological traits that vary across individuals and remain relatively stable over time^15,22–27^, including across the adult lifespan^29^. These features show moderate heritability—typically 30–60%, with particularly strong genetic influence in unimodal cortical systems such as sensorimotor and visual regions^30^—yet they also reflect environmental and developmental shaping. Variations in network size may offer insight into underlying neural mechanisms: larger networks may represent compensatory recruitment in response to processing inefficiencies, whereas smaller networks may indicate reduced engagement associated with symptom severity or cognitive impairment. Evidence supports compensatory enlargement in attentional systems, especially the dorsal attention network (DAN), where studies demonstrate hyperactivity and increased recruitment during simple detection tasks and high-demand attentional paradigms^31,32^, often interpreted as a response to underlying inefficiencies^33–36^. Against this neurobiological backdrop, assessing individual-level network size and topographic organization provides a promising avenue for characterizing disease-related alterations. One emerging method—Functional Network Comparative Area and Topography Analysis (FUNCATA)—quantifies these variations in network structure across individuals^30,37^. By focusing on network area rather than rigid anatomical boundaries or predefined connectivity metrics, FUNCATA captures subtle topographic abnormalities that may be especially relevant in psychiatric disorders^37,38^. This individualized approach offers a mechanistic framework for identifying biologically grounded disruptions in schizophrenia and may ultimately support the development of more precise biomarkers and targeted interventions.

In prior work, we examined network size and topography in schizophrenia and bipolar disorder (*n* = 27 and *n* = 35, respectively), representing the first application of the FUNCATA approach to schizophrenia research, and found enlarged Dorsal Attention Network (DAN) and Language Network (LAN) in schizophrenia^37^. DAN size correlated with mood dysregulation, and LAN enlargement was linked to psychotic-like experiences in healthy participants from the Human Connectome Project – Young Adult (HCP-YA) dataset, suggesting LAN enlargement may be a marker of psychosis vulnerability^37^. Other studies have reported similar findings in different disorders, such as enlarged Salience Network (SN) in major depression^39^. Using FUNCATA, we also observed SN enlargement, along with enlarged left-sided Default Mode Network (DMN) and Cingulo-Opercular Network (CON), in a patient with major depression and maladaptive daydreaming^38^. These findings support FUNCATA’s utility in identifying meaningful network alterations across clinical and subclinical spectra and suggest some network changes may be disorder-specific.

Building on our previous findings, the current study aimed to replicate and expand results related to functional network enlargement in schizophrenia. We hypothesized that individuals with schizophrenia would show increased DAN and LAN size relative to healthy controls. To test this, we analyzed data from 86 individuals with non-affective psychotic disorders (NAP) in the Human Connectome Project – Early Psychosis (HCP-EP) dataset. This study extends our prior work in several ways. First, we examined 10 networks (vs. 8 previously), adding the SN and auditory network (AUD), to capture alterations in circuits increasingly implicated in psychosis, including salience processing^40^ and aberrant auditory perception^41^. Second, we evaluated hemispheric effects because lateralized disturbances—including language asymmetry reductions and hemispheric specialization abnormalities—are well-documented features of schizophrenia and may reveal additional pathophysiological signatures^42,43^. Third, we expanded our characterization of network topography by incorporating an additional metric—a topographic abnormality index—that quantifies the degree of deviation in vertex-level network assignment relative to normative patterns, complementing our probability-based maps. Together, these enhancements offer deeper insight into large-scale brain network alterations in schizophrenia and contribute to understanding its neural basis.

## Methods

We analyzed data from the Human Connectome Project – Early Psychosis (HCP-EP) study^44^, Release 1.1 (10.15154/1522899), comprising 143 participants aged 18–34: 86 with non-affective psychosis (NAP) and 57 healthy controls (HC). Affective psychosis participants were excluded. The numerical imbalance between the NAP and HC groups reflects the HCP-EP consortium’s intentional recruitment strategy, which prioritized oversampling individuals with psychosis to ensure adequate representation of diagnostic heterogeneity and sufficient power for detecting illness-related effects. Despite this imbalance, the HC sample (*n* = 57) is comparable to or larger than those used in functional network and resting-state fMRI studies of schizophrenia and early psychosis (e.g.^45–48^,). To ensure that group size differences did not bias connectivity or topographic estimates, we conducted sensitivity analyses involving: a) covariate-adjusted analyses controlling for age, sex, and in-scanner motion (frame displacement, FD), and b) group-matched subsampling analyses based on age and FD. The study was conducted across sites in Massachusetts (Beth Israel Deaconess, McLean, MGH), Indiana (Indiana University), and coordinated at Brigham and Women’s Hospital.

NAP participants included individuals diagnosed with schizophrenia (*n* = 60), schizoaffective disorder (*n* = 12), schizophreniform disorder (*n* = 9), or other psychotic disorders (*n* = 5). Inclusion/exclusion criteria, previously described^44,49^, excluded participants with substance-induced psychosis, psychotic disorder due to medical condition, IQ < 70, HIV, significant neurological disorder, MRI contraindications, recent ECT, severe substance use disorder (past 90 days), active suicidality, or high aggression risk. Informed consent was obtained; protocols were IRB-approved at all sites.

### Behavioral assessments

Diagnoses were confirmed via the Structured Clinical Interview for DSM-5-RV^50^ and clinical data. Psychotic symptoms were assessed with the PANSS using Marder’s 5-factor model to derive positive, negative, and disorganized scores^51,52^. Depression and mania were assessed using the Montgomery-Asberg Depression Rating Scale^53^ and Young Mania Rating Scale^54^, respectively.

Cognitive performance was measured using NIH Toolbox and the Penn Computerized Neurocognitive Battery. Administered tests included episodic memory (Picture Sequence Memory), executive function (Dimensional Change Card Sort), language (Oral Reading Recognition and Picture Vocabulary), processing speed (Pattern Comparison), working memory (List Sorting), and auditory function (Words-in-Noise). Emotion recognition was evaluated using the Penn Emotion Recognition Test^55^. Raw scores were used in all analyses.

### MRI acquisition

Imaging was conducted on 3 different 3 T Siemens Prisma scanners with a 32-channel head coil (2 scanners) or a 64-channel head/neck coil (1 scanner). T1-weighted structural images were acquired using 3D MPRAGE (0.8 mm isotropic, TR/TI = 2400/1000 ms, TE = 2.22 ms, flip = 8°, FOV = 256 × 240 × 166 mm, matrix size = 320 × 300, 208 sagittal slices, in-plane (iPAT) acceleration factor of 2). T2w volumes were obtained using 3D SPACE sequence (TR/TE = 3200/563 ms). Four 5.8 min resting-state functional scans (420 whole-brain volumes) were collected using 2 mm isotropic resolution, multiband acceleration factor of 8, TR 800 ms, TE 37 ms, 52-degree flip angle, 72 2-mm slices, and a 208-mm in-plane field-of-view. Two scans were acquired with A > P phase encoding.

### Image preprocessing

Functional MRI data from all participants were processed using HCP minimal pipelines (v4.4.0)^56^ inside the QuNex container (v0.93.2)^57^. Subsequently, CIFTI-format resting-state “grayordinate” data (gray matter surface vertices plus subcortical gray matter voxels, in a standardized, aligned space) underwent denoising using FMRIB’s ICA-based X-noiseifier (FIX)^58,59^. For this denoising, we first regressed out 24 motion-parameter confounds (*C*_*m*_; 6 rigid body motion parameters, their temporal derivatives, and the squares of the 12 resulting regressors) from both the data *(Y)* and time series of the ICA components:

$${Y}_{m}=Y-{C}_{m}\cdot ({pinv}({C}_{m})\cdot Y),$$

$${{\rm{I}}{CA}}_{m}={\rm{I}}{CA}-{C}_{m}\cdot ({pinv}({C}_{m})\cdot {ICA}),$$

where the row dimension of each variable is timepoints and *pinv* is the pseudoinverse.

Then, the motion-cleaned ICA component time series (*ICA*_*m*_) was regressed onto the motion-cleaned data (*Y*_*m*_):

$${\beta }_{{ICA}}={pinv}({{ICA}}_{m})\cdot {Y}_{m}$$

Finally, the subset of motion-cleaned ICA components corresponding to the “noise” components identified by FIX (*ICA*_*m*_*(:,noise)*) were removed from the motion-cleaned data to yield the FIX-cleaned data:

$${Y}_{{clean}}\,={Y}_{m}-{{ICA}}_{{\rm{m}}}\left(:,{noise}\right)\cdot {\beta }_{{ICA}}({noise},:)$$

Multimodal surface alignment (MSMAll) was used to register cortical surfaces using sulcal depth, myelin maps, functional connectivity, and visuotopy^60,61^. Then using custom code (outside QuNex) the mean grayordinate timecourse across all grayordinates was regressed from the timeseries of each grayordinate (i.e., the grayordinate analog of “global signal regression”)^62^, followed by temporal band-pass filtering (0.009–0.08 Hz). The four runs were concatenated, and a 6 mm FWHM surface-based smoothing applied.

### Functional network template matching

**Fig. 1:**
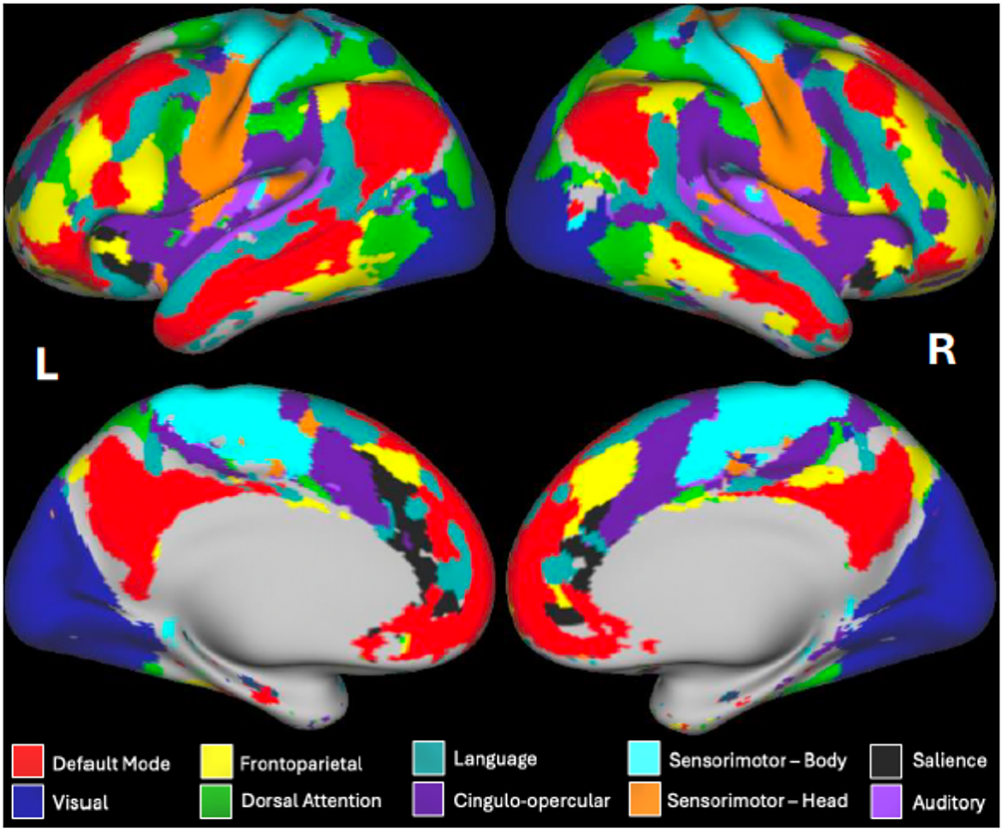
Functional connectivity networks in a healthy participant. The figure shows a colorized brain map of ten resting-state functional connectivity networks in a sample healthy participant derived from the individual-specific template matching procedure^63^. Areas in gray represent vertices whose connectivity profiles best matched to two networks (i.e., the cingulo-parietal network and the retrosplenial temporal network) that were not included in the present study due to their small size in the group-based template maps.

The template-matching procedure for deriving individual-specific functional network assignments has been previously described^37,63^. Briefly, a set of network templates were generated by assigning the surface vertices to functional networks based on independent group-average connectivity data^24,63^. We then assigned individual-specific network labels to each vertex (from among a set of 10 group-average networks) for the participants in the present study by matching each vertex’s individual-specific connectivity pattern to the set of templates. Two additional networks (i.e., salience and auditory) were included in the present study, which were excluded from our earlier study^37^ due to their relatively small size. The matching process first correlates the timecourse of each vertex with the timecourses of all other vertices in that participant’s data, yielding a connectivity map for each vertex, which was Fisher z-transformed, and then thresholded and binarized to the top 5% of connectivity values (across vertices). This results in a binarized map of vertices with high connectivity to the reference cortical vertex. The reference vertex is then assigned to the network from the group templates with the highest similarity (Dice coefficient) to the binarized connectivity map for that vertex in that individual. This process is then repeated separately for each vertex, yielding an individualized network map across the cortical surface. An example is shown in Fig. 1. Subcortical grayordinates did not contribute to the template matching process.

### Statistical analysis

Statistical analyses were done using SAS 9.4 (SAS Institute Inc., Cary, NC). Network size was defined as the number of surface vertices assigned to each network, normalized to total surface vertices. Group comparisons (NAP vs. HC) were assessed using ANCOVA, with and without adjusting for age and sex. Spatial maps of regions with a different rate of assignment to each network between groups (NAP vs. HC) were generated using a chi-square test for differences in proportions between the two groups at each surface vertex. To control for multiple comparisons across the surface vertices, a previously described cluster-size correction was implemented via permutation testing^64^. Specifically, we randomly permuted the participant labels (NAP vs. HC) 1000 times, computed proportion maps for each network for each permutation per the same template matching procedure outlined above and then converted those maps to clusters of vertices having a vertex-wise *p*-value < 0.05 in a chi-square test for differences in proportions. The test statistic for each permutation was the number of vertices in the largest cluster. Those cluster sizes were stored for each permutation to create a distribution of cluster sizes for each network under the null (permutation-based) distribution. Clusters in the true NAP vs. HC comparison were considered significant if their size exceeded the 95th percentile of this null distribution.

**Fig. 2:**
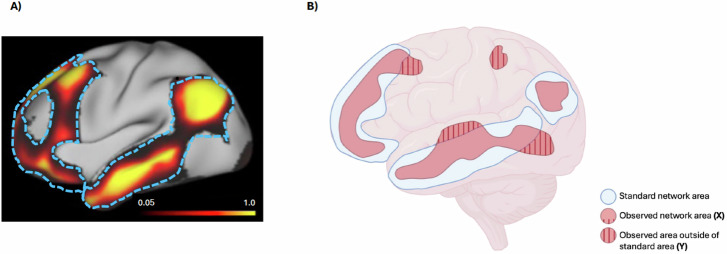
Derivation of the Topographic Abnormality Index (TAI). Panel (**A**) shows a surface map of the proportion of individuals from HCP-YA (*N* = 1003) in which the underlying vertex was assigned to the default mode network (left lateral surface only) by the template matching procedure, excluding regions where the network is localized in less than 5% of participants. Bright yellow indicates regions where the default mode network was assigned in close to 100% of participants, and black/dark red regions indicate a much lower probability of default mode network at that location across HCP-YA individuals. Dashed blue lines delineate the probability map and represent borders of the “standard network area” as used in the TAI calculation. Panel (**B**) is a schematic representation of the Topographic Abnormality Index (TAI) in the default mode network. Blue regions represent the standard network area, and red regions (both plain and striped) are the areas assigned to the default mode network (**X**) of a hypothetical participant, with some regions (striped) extending beyond the standard network area border (**Y**). The TAI is calculated as Y/X. Areas were operationalized as the number of vertices.

We also assessed the severity of each functional network’s Topographic Abnormality Index (TAI), as depicted in Fig. 2. The TAI represents the proportion of a network’s label that extends beyond a liberal (generous) definition of the vertices associated with that network in a normal population, defined as the vertices with at least 5% representation in that network in the population from the Human Connectome Project - Young Adult dataset^37^. Because these standard regions were derived from this independent HCP–Young Adult population rather than from the healthy controls in our study, the reference provides an unbiased external benchmark for computing TAI. The TAI therefore measures the fraction of the labelled vertices within a given network that fall outside this standard area, and was calculated using the formula:

$${\rm{Topographic\; Abnormality\; Index}}\left({\rm{TAI}}\right)=\frac{\left|A-B\right|}{\left|A\right|}$$

where *A* is the number of vertices in the individual’s network (per template matching), *B* is the number of vertices in the standard region, and ∣A − B∣ counts how many vertices are *outside* the standard region. A value of 0 would mean that all vertices are within the standard region and 1 would mean all voxels are outside the standard region.

Linear regression was used to examine associations between brain networks and behavioral predictors, including symptom dimensions (positive, negative, disorganized, depression, mania), antipsychotic exposure (chlorpromazine equivalent, duration), and cognitive measures (executive function, working memory, emotion recognition, vocabulary, processing speed, reading decoding). Stepwise regression (*p* = 0.05 for entry/removal) identified the most relevant predictors. Given the number of clinical and cognitive association tests performed, false discovery rate (FDR) correction using the Benjamini–Hochberg procedure was applied separately to functional network size and TAI analyses to control for multiple comparisons.

To define psychosis-related network biotypes, we applied k-means clustering to functional network sizes in NAP participants, grouping individuals based on Euclidean distance. The optimal number of clusters was determined using the highest Cubic Clustering Criterion (CCC). Demographic and clinical features were then compared across sub-groups.

Effect sizes were calculated using Cohen’s d, which measures standardized group differences. Values of 0.2, 0.5, and 0.8 reflect small, medium, and large effects, respectively^65^.

## Results

### Demographic and clinical profile

Table 1 shows demographic and clinical information across the participant groups. There were roughly twice as many males than females in the study sample.

**Table 1:** Demographic and clinical characteristics of healthy control and non-affective psychosis subjects.

Characteristic	Control (*n* = 57)	NAP (*n* = 86)	t/χ^2^	*p*
**Age** (s.d.)	24.3 (4.2)	21.7 (3.3)	4.1	<0.0001
**Sex** (%)			0.2	0.6
Female	20 (35.1)	27 (31.4)		
Male	37 (64.9)	59 (68.6)		
**Ethnicity** (%)			24.5	<0.0001
Asian	8 (14.0)	5 (5.8)		
Black	5 (8.8)	41 (47.7)		
White	41 (71.9)	36 (41.9)		
Mixed	1 (1.8)	1 (1.2)		
Other	2 (3.5)	3 (3.5)		
**Chlorpromazine**				
**Equivalent,** **mg/d** (s.d.)	-	206.5 (242)	n/a	n/a
**Antipsychotic**				
**Exposure,** **months** (s.d.)	-	15.1 (15.4)	n/a	n/a
**Framewise**				
**Displacement, mm** (s.d.)	0.117 (0.04)	0.160 (0.09)	3.74	0.0003

### Functional network size in psychosis

**Fig. 3:**
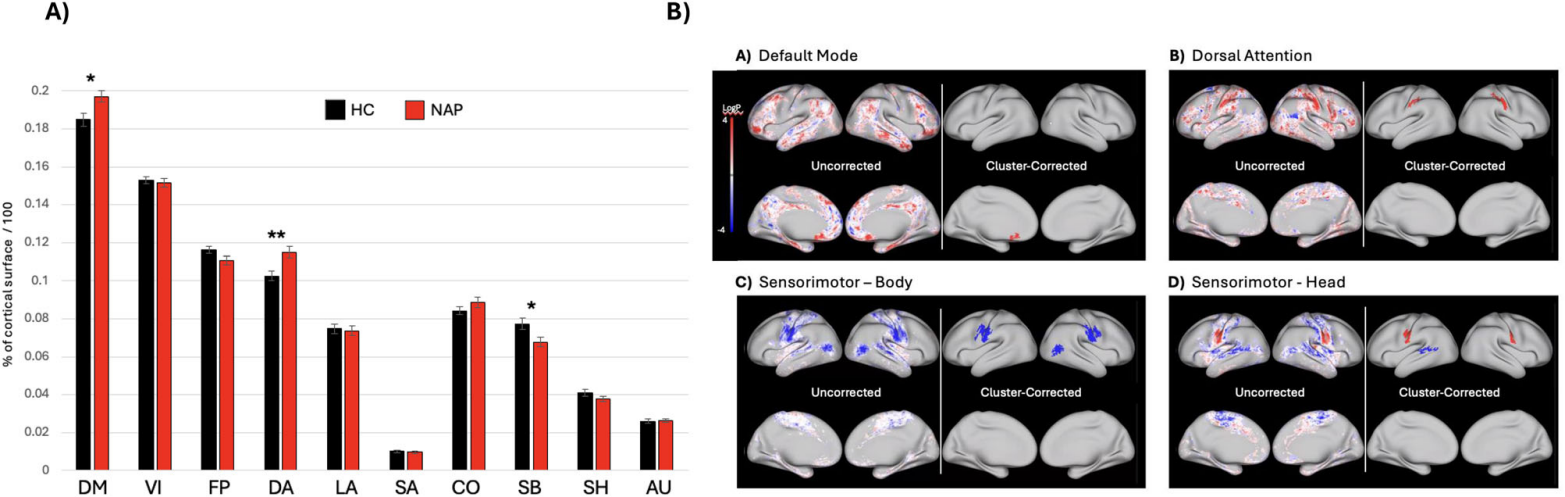
Size and topography of functional connectivity networks in non-affective psychosis and healthy control participants. Panel (**A**) compares the average relative network size of ten functional networks (relative to the total number of cortical surface vertices) across non-affective psychosis (*n* *=* 86; red bars) and healthy control (*n* *=* 57; black bars) participants from the Human Connectome Project – Early Psychosis study. Error bars represent standard error. DM default mode network, VI visual network, FP frontoparietal network, DA dorsal attention network, LA language network, SA salience network, CO cingulo-opercular network, SB sensorimotor-body network, SH sensorimotor-head network, AU auditory network. Images in (**B**) are the results of chi-square tests at each surface vertex, comparing the proportion of individuals assigned to the network at that vertex in non-affective psychosis participants (*n* *=* 86) to the proportion in healthy control participants (*n* *=* 57). Color-coding is based on log_10_(*p*-value) values. Red shading indicates vertices where the network localizes more frequently in NAP participants. Blue shading indicates vertices where the network localizes more frequently in control participants. Right side within each panel indicate significant clusters (*p* *<* 0.05) after permutation-based cluster-size correction. Only results for the four networks showing significant regional localization after cluster-size correction are shown. (Results for other networks can be found in Supplemental Fig. 2).

Mean (±s.d.) sizes of the ten functional networks in HC and NAP groups, relative to total cortical size, are shown in Fig. 3A and Supplemental Table 1. A significant group difference was found in the dorsal attention network (DAN), with NAP at 11.5% (0.03%) and HC at 10.3% (0.02%) (F = 7.99, *p* = 0.005), yielding a moderate effect (Cohen’s d = 0.48, 95% CI [0.13, 0.92]). This difference remained after adjusting for age and sex (F = 5.64, *p* = 0.02). Post hoc tests showed similar effects in both hemispheres (Supplemental Fig. 1).

No significant group differences were found in the size of the language network (LAN) (F = 0.07, *p* = 0.6), even after adjusting for age and sex (F = 1.7, *p* = 0.8). However, significant differences were observed in the default mode network (DMN) and sensorimotor-body network (SBN). DMN size was larger in NAP (19.7% ± 0.03%) than HC (18.5% ± 0.03%) (F = 6.31, *p* = 0.013; d = 0.43, 95% CI [0.08, 0.85]). SBN size was smaller in NAP (6.8% ± 0.02%) than HC (7.7% ± 0.03%) (F = 5.32, *p* = 0.022; d = 0.39, 95% CI [0.06, 0.65]).

Group differences remained significant after adjusting for mean frame displacement (FD) for the DAN (F = 6.15; 0.01), DMN (F = 7.32; *p* = 0.008) and SBN (F = 6.57; *p* = 0.01).

We conducted complementary sensitivity analyses using subgroup restriction to further address between-group differences in age and head motion. Specifically, the NAP group was reduced to match the control sample size (*n* = 57 per group) using two independent approaches: (i) exclusion of the youngest NAP participants to match age distributions between groups, and (ii) exclusion of NAP participants with the highest FD to match motion levels between groups. Across both subgrouping strategies, patterns of functional network size differences were highly similar to those observed in the full sample, indicating that the network size findings were robust to differences in age distribution and in-scanner motion (Supplemental Tables 2 and 3).

### Topography of functional networks in psychosis

Figure 3B shows uncorrected and cluster-corrected differences in network surface patterns between NAP and control groups, based on vertex-wise network assignment probabilities. After permutation-based cluster-size correction, significant group differences emerged in four networks: 1) DMN (left subgenual cingulate cortex), 2) DAN (somatosensory cortex), 3) SBN (inferiolateral sensorimotor and right lateral occipitotemporal cortices), and 4) SHN (inferiolateral sensorimotor and left posterior superior temporal cortices). In some areas (DMN, DAN, SHN; red), networks were more commonly assigned in NAP than controls, and less so in others (SBN, SHN; blue), as shown in Fig. 3B.

Notably, inferiolateral sensorimotor cortex was more frequently assigned to SHN in NAP, but to SBN in controls—highlighting a “push/pull” relationship between SHN and SBN assignment. This shift suggests that in NAP, the region was more often associated with the “canonical” network for that area (SHN). Uncorrected and corrected maps for remaining networks appear in Supplemental Fig. 2.

### Topographic Abnormality Indexes (TAI) of functional networks

**Fig. 4:**
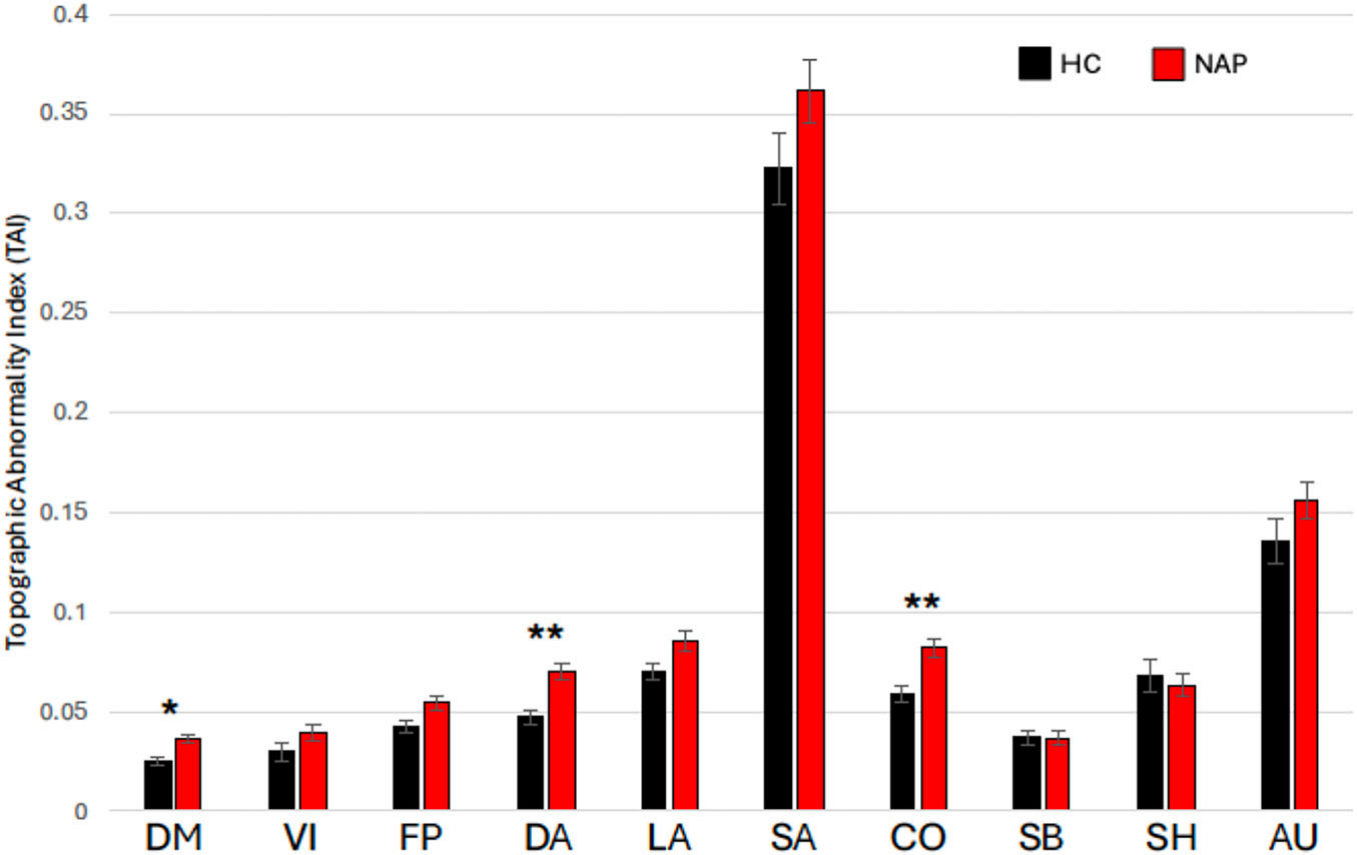
Topography Abnormality Indexes (TAI) of functional networks in psychosis and control participants. Figure shows mean TAI for non-affective psychosis (red bars) and healthy control (black bars) participants for ten functional networks. See Fig. 3 caption for network labels. *p < 0.005. **p < 0.0005.

Mean Topographic Abnormality Indexes (TAI) for the ten functional networks in NAP and controls are shown in Fig. 4 and in Supplemental Table 4. Notably, mean TAI values were inversely related to network size, with smaller networks showing higher TAI values. After Bonferroni correction, significant group differences were found in the DMN (F = 11.6, *p* = 0.0009; d = 0.13), DAN (F = 12.9, *p* = 0.0005; d = 0.23), and CON (F = 13.9, *p* = 0.0003; d = 0.44), with higher TAI in NAP participants. These differences remained significant after adjusting for age and sex: DMN (*p* = 0.0007), DAN (*p* = 0.0006), and CON (*p* = 0.0002). Group differences remained significant after adjusting for mean frame displacement (FD) for the DMN (*p* = 0.005), DAN (*p* = 0.01) and the CON (*p* = 0.009).

Sensitivity analyses using subgroup restriction yielded largely similar results. When the NAP group was reduced to match controls by age, TAI differences closely mirrored those observed in the full sample (Supplemental Table 5). When the NAP group was instead restricted to balance framewise displacement, group differences in DMN, DAN, and cingulo-opercular TAI remained statistically significant, although effect sizes and *p* values were attenuated (Supplemental Table 6). This pattern suggests that topographic abnormalities are more sensitive to head motion than network size measures, while remaining detectable after motion balancing.

### Sub-group analysis of functional networks in NAP

**Fig. 5:**
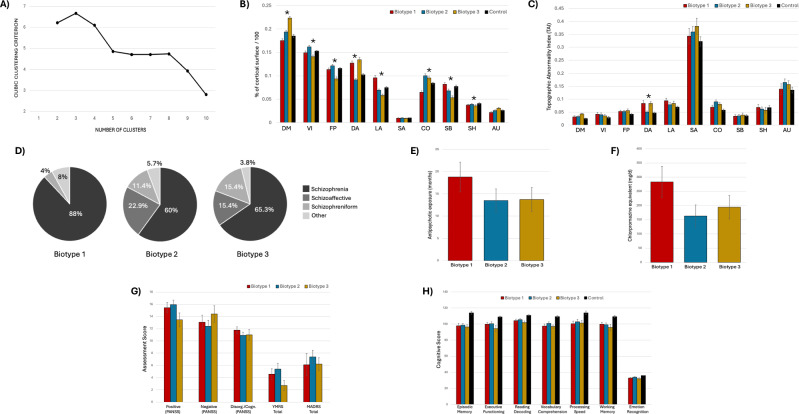
Functional network size-derived biotypes in psychosis with associated network and clinical features. Graph (**A**) shows that the Cubic Clustering Criterion identified three clusters as the optimal solution following k-means clustering, used to define data-driven ‘biotypes’ based on patterns of network size differences among participants with non-affective psychosis. Panel (**B**) shows the mean relative network sizes across the three psychosis biotypes and healthy controls. See Fig. 3A caption for network labels. Error bars represent standard error. Panel (**C**) shows the mean network Topographic Abnormality Indexes (**C**) across groups. The other panels display the clinical profiles associated with each biotype, including: (**D**) psychotic disorder diagnoses; (**E**) lifetime antipsychotic medication exposure, in months; (**F**) chlorpromazine equivalent dose of the current antipsychotic medication; (**G**) clinical symptomatology, including positive, negative and disorganization symptoms (derived from the Positive and Negative Syndrome Scale, PANSS), mania symptoms (derived from the Young Mania Rating Scale, YMRS), and depressive symptoms (derived from the Montgomery-Asberg Depression Rating Scale, MADRS); and (**H**) neurocognitive functioning scores (derived from the NIH Toolbox, with the exception of emotion recognition which was derived from the Pennsylvania Neurocognitive Battery). Asterisks in panels **B** and **C** denote variables showing a significant omnibus group effect across the three psychosis biotypes after false discovery rate (FDR) correction within each domain (*q* < 0.05). Group differences in panels **E** and **F** were not significant (*p* > 0.05). No clinical or cognitive measures in panels **G** and **H** survived FDR correction. Error bars indicate standard error.

A k-means cluster analysis of functional network sizes in NAP participants identified three distinct clusters or biotypes (Fig. 5A, B). Among the 86 individuals, 25, 35, and 26 were classified into Biotypes 1, 2, and 3, respectively. Biotype 1 showed enlarged DAN and LAN and a smaller CON compared to controls. Biotype 2 closely resembled the control group’s network profile, indicating minimal deviation. Biotype 3 had enlarged DMN and DAN and smaller FPN and SBN. TAI values across size-derived biotypes (Fig. 5C) revealed that patterns of topographic deviation often paralleled network enlargement, most prominently in the DAN and CON. Notably, TAI was consistently lower in controls than in each of the three biotypes across most functional networks, suggesting that TAI may serve as a more sensitive indicator of illness-related alterations than network size.

Demographic characteristics across biotypes were similar in age, sex, and ethnicity (Supplemental Table 7). Biotype 1 had the highest schizophrenia rate (88%) and no schizoaffective cases (Fig. 5D), as well as the longest treatment duration (Fig. 5E) and highest antipsychotic dose (Fig. 5F); however, these differences were not statistically significant. Biotype 2 showed higher mood scores (YMRS, MADRS), while Biotype 3 had low positive and high negative symptoms (Fig. 5G), although these differences did not survive FDR correction. Cognitive functioning was lower in all NAP biotypes compared to healthy controls (Fig. 5H), but these effects were likewise not significant after FDR correction.

### Clinical and cognitive relationships to functional network size

Stepwise regression was conducted for the three networks showing significant group differences in size. For the DMN, three predictors emerged: 1) mania, 2) antipsychotic duration, and 3) working memory. DMN size was negatively associated with mania (R² = 0.1346, F = 11.82, *p* = 0.001), duration of antipsychotic use (partial R² = 0.0574, model R² = 0.1920, F = 5.33, *p* = 0.0237), and working memory (partial R² = 0.0434, model R² = 0.2354, F = 4.2, *p* = 0.0439). After FDR correction across all network size association tests, the associations between DMN size and mania (q = [0.0035]), antipsychotic duration (q = [0.0332]) and working memory (q = [0.0439]) remained significant.

No significant predictors were found for DAN overall. However, when analyzed by biotype, DAN size was significantly associated with: 1) depression in biotype 1 (R² = 0.1876, F = 4.9, *p* = 0.039, q = 0.0455), 2) executive functioning in biotype 2 (R² = 0.2585, F = 9.8, *p* = 0.004, q = 0.0093), and 3) processing speed in biotype 3 (R² = 0.2585, F = 6.4, *p* = 0.018, q = 0.0315). All three associations survived FDR correction (q < 0.05)

For the SBN, working memory was the only significant predictor, showing a positive association with network size (R² = 0.1452, F = 12.9, *p* = 0.0006, q = 0.0042).

Supplemental Fig. 3 illustrates correlations between these clinical and cognitive measures and functional network size both across the full psychosis cohort and within individual biotypes.

### Clinical and cognitive relationships to network topographic abnormality index (TAI)

For DMN TAI, stepwise regression identified three significant cognitive predictors. Greater topographic abnormality was associated with poorer vocabulary comprehension (R² = 0.104, F = 8.34, *p* = 0.005), slower processing speed (partial R² = 0.068, model R² = 0.17, F = 5.85, *p* = 0.018), and lower emotion recognition performance (partial R² = 0.053, model R² = 0.23, F = 4.76, *p* = 0.033). All three associations remained significant after FDR correction (vocabulary comprehension: q = 0.0150; processing speed: q = 0.0360; emotion recognition: q = 0.0396).

For DAN TAI, two significant predictors emerged: higher antipsychotic dose (R² = 0.16, F = 13.81, *p* = 0.0004) and lower depression severity (MADRS score; partial R² = 0.06, model R² = 0.29, F = 5.3, *p* = 0.024). Both associations survived FDR correction (antipsychotic dose: q = 0.0024; depression severity: q = 0.0360).

For CON TAI, the only significant predictor was PANSS disorganization, where higher disorganization scores were associated with greater topographic abnormality (R² = 0.057, F = 4.36, *p* = 0.04). This association also remained significant after FDR correction (q = 0.040),

## Discussion

### Replication of prior findings and network size abnormalities in psychosis

Our replication study assessed functional network size and topography in individuals at an early stage of psychotic disorder. In our earlier work on schizophrenia, using the same methodology and healthy controls from the HCP-Young Adult study, we observed enlargement of the dorsal attention network (DAN) and language network (LAN) in schizophrenia but not in bipolar disorder. DAN size was also linked to mood dysregulation severity^37^.

Our replication study found increased DAN size in individuals with non-affective psychotic disorders. The DAN was about 11.6% larger in the psychosis group compared to controls, with a moderate effect size (d = 0.48), slightly less than the 16% difference seen in our earlier schizophrenia study^37^. While the interpretation remains uncertain, larger networks may indicate greater neural resource recruitment for associated functions. As the DAN supports top-down attentional control—enhancing relevant stimuli and suppressing distractions^66,67^—its enlargement could reflect compensatory restructuring to offset attentional or cognitive control deficits. Supporting this, previous work has shown DAN overactivity in schizophrenia during both basic target detection^31^ and tasks requiring high attentional demand^32^. Therefore, an enlarged DAN may stem from compensatory hyperactivity or hyperconnectivity, potentially causing inefficient or noisy processing during attentional tasks. Our consistent finding of DAN enlargement across studies highlights its promise as a biomarker or risk predictor for schizophrenia.

### Novel network size findings and implications for illness stage

We did not find enlarged LAN in non-affective psychosis, unlike in our earlier schizophrenia study. This may be due to different case populations: the prior study included schizophrenia patients regardless of illness stage, while the current sample involved early-stage psychotic disorders, including shorter-duration cases like schizophreniform disorder. LAN enlargement might be specific to chronic or severe psychoses, reflecting illness progression or antipsychotic exposure. In this study, enlarged LAN was seen only in psychosis biotype 1, which had the highest proportion of schizophrenia (88%) and greatest antipsychotic dose. These LAN abnormalities matched those in our earlier work. Our LAN maps mostly cover superior temporal cortex and a smaller dorsolateral prefrontal region near Broca’s area. Previous research in antipsychotic-naïve patients found connectivity changes in superior temporal areas only in longer illness duration^68,69^. Thus, LAN enlargement may indicate illness chronicity, consistent with prior findings of superior temporal dysconnectivity^68^.

We also found that in psychosis patients, the default mode network (DMN) was larger, and the sensorimotor-body network (SBN) was smaller than in controls. These differences were not seen in our earlier schizophrenia study^37^, likely due to differing clinical characteristics across cohorts. DMN enlargement may reflect early-stage network restructuring or lower symptom severity. This is supported by its presence in psychosis biotype 3, which included the highest proportion of patients with shorter-duration schizophreniform disorder and relatively mild psychotic or manic symptoms. The DMN is active during rest and supports internally directed processes like daydreaming, self-reflection, and imagining the future^70,71^. Studies in schizophrenia have reported altered DMN connectivity, especially increased activity in the medial prefrontal cortex and posterior cingulate/precuneus, which may underlie excessive internal focus and reduced cognitive control^72,73^, even early in illness^74,75^. Increased DMN connectivity has also been seen in clinical high-risk populations^76–79^, supporting its role early in psychosis. Meta-analyses show both increased and decreased DMN connectivity across contexts, reflecting heterogeneity in its functional role in schizophrenia^14^, a pattern echoed in our results. Although rarely studied in terms of size, DMN enlargement may not be specific to psychosis. Our earlier case report of a patient with depression and maladaptive daydreaming showed left-sided DMN enlargement^38^. This suggests DMN size may reflect general internally focused cognition, not a psychosis-specific change, emphasizing the need to include transdiagnostic factors like mood in future network-based models of psychopathology.

The significance of the reduced SBN size in psychosis remains unclear and may be an “epiphenomenon” due to controls more often having inferolateral sensorimotor cortex assigned to SBN in this dataset, even though that region is typically linked to SHN in group-based maps. Nonetheless, sensorimotor abnormalities—such as motor coordination issues, sensory gating deficits, and altered proprioception—are common in schizophrenia and the prodrome, reflecting dysfunction in motor and sensory cortices^80–82^. These issues may stem from disrupted connectivity and structural changes in the broader sensorimotor cortex (i.e., SBN/SHN complex).

### Biotypes reveal heterogeneous network size patterns

Our study examined the heterogeneity of functional network size among psychosis patients and identified three distinct patterns. One group (Biotype 1) showed a network profile closely matching our prior findings in schizophrenia, with enlarged LAN and DAN^37^. This subgroup also had a higher proportion of schizophrenia diagnoses, greater symptom severity, and greater cumulative antipsychotic exposure; however, these clinical differences are reported for descriptive purposes only, as they did not reach statistical significance. Biotype 2 had network sizes most similar to healthy controls and included a higher proportion of individuals with mood-related symptoms and schizoaffective diagnoses, though these differences were likewise not statistically significant. Biotype 3 was marked by enlarged DMN and DAN, and included more individuals with shorter illness duration, possibly reflecting early illness-related changes.

### Clinical associations with network size

Few clinical relationships with functional network size were observed, and none involved the DAN across all psychosis participants—possibly due to clinical heterogeneity. However, exploratory analyses within biotypes revealed that DAN size correlated with depression severity in Biotype 1, executive functioning in Biotype 2, and processing speed in Biotype 3. These findings align with literature linking executive function and processing speed to attentional control, a key role of the DAN^66,67^. A mood-related relationship with DAN size was also found in our earlier study^37^, though the direction differed—here, greater depression related to a smaller DAN. While the DAN is not directly involved in mood regulation, its interactions with the DMN and salience network (SAL) may influence emotional states^83,84^. A smaller DAN with more depressive symptoms could reflect a shift of resources toward mood-related networks like the DMN or SAL^39,85^. Across the full psychosis sample, higher antipsychotic use was linked to smaller DMN size, possibly indicating medication-related normalization of DMN connectivity^85,86^. Lastly, better working memory with smaller DMN size supports the idea that reduced DMN activity enhances task-positive function and may reflect improved task switching^87^.

### Topographic abnormalities and network-specific patterns

We examined topographic differences in functional networks using two main approaches. The vertex-wise method identified cortical regions more frequently linked to each network in psychosis. Only four networks—DMN, DAN, SBN, and SHN—showed significant group differences after cluster-size correction, suggesting cortical dysconnectivity patterns in psychosis. The most notable differences were in the SBN. Psychosis participants were less likely to have inferiolateral sensorimotor cortex assigned to SBN and more likely to have it assigned to SHN, its canonical network. They were also less likely to have lateral occipitotemporal cortex assigned to SBN. This region, involved in processing visual information about objects, bodies, and actions^88^, is not canonically SBN but is sometimes assigned to it via template matching—more often in healthy controls. This occasional SBN assignment may suggest a role in integrating visual and sensorimotor information in some individuals. Overall, the SBN differences may relate to motor coordination and sensorimotor processing deficits in schizophrenia^89^.

The second approach used the Topographic Abnormalities Index (TAI) to quantify topographic disruption. This analysis showed that DMN and DAN extend beyond typical boundaries in psychosis. The cingulo-opercular network (CON) also showed elevated TAI in psychosis despite having a normal overall size, indicating a spatial shift in vertex distribution. While no CON regions survived cluster correction, several uncorrected areas showed group differences (see Supplemental Fig. 2). CON hypoconnectivity has been reported in schizophrenia^90^ and in relation to psychotic experiences^37,91^, and may reflect disruptions in goal-directed behavior^92^ and salience processing^93^, contributing to distractibility and impaired reality testing. Regression analyses using the TAI indicate that topographic abnormalities may have clinical relevance. Higher DMN TAI was linked to cognitive impairment, consistent with the DMN’s diverse cognitive roles due to its broad cortical distribution^94^. DAN topographic abnormality was most strongly associated with antipsychotic dose, potentially reflecting illness severity. The inverse relationship between DAN TAI and depression severity is unclear but could reflect treatment effects or differences in illness profile, as non-schizophrenia participants in the psychosis group tended to report more depressive symptoms. Lastly, higher CON TAI was associated with greater disorganization—a symptom predictive of psychosis conversion in at-risk individuals^95,96^—suggesting that CON topography may have prognostic value.

### Integration of network size and topography metrics

Although network size and TAI were analyzed separately, these measures capture related yet distinct dimensions of functional architecture. Network size reflects the overall spatial extent of a system, whereas TAI quantifies the degree to which that extent deviates from normative boundaries. Their inverse association suggests that larger networks more often extend beyond canonical limits, while smaller networks may show more focal spatial shifts. This integration helps explain why both metrics converge on abnormalities in the DAN and DMN in psychosis, highlighting that disruptions in schizophrenia span multiple spatial dimensions of network organization. Notably, the CON showed elevated TAI despite normal size, indicating that some networks may manifest illness-related pathology primarily through altered spatial configuration rather than global expansion. This pattern suggests that TAI—and other shape-based metrics—may detect subtle forms of disorganization that are not captured by size alone. Consistent with this interpretation, controls demonstrated lower TAI than each of the three biotypes across most functional networks, pointing to the possibility that TAI may represent a more sensitive indicator of illness-related alterations—and potentially a more robust determinant of psychopathology—than network size.

### Limitations and future directions

Our study has some limitations. Firstly, while we identified functional network size and topographic abnormalities in psychosis—most consistently involving the DAN—some variability likely reflects measurement noise. However, sensitivity analyses accounting for framewise displacement indicated that network size differences were preserved, whereas topographic abnormality effects were attenuated, suggesting that network size may represent a more robust marker than topographic measures. Secondly, with an effect size near 0.5, DAN size alone may not serve as a diagnostic tool but could be useful as part of a multi-biomarker panel or for risk stratification. For predicting treatment response, relapse, or conversion to psychosis, combining it with other predictors may have clinical value. The utility of our topographic metrics for risk prediction should be tested in longitudinal studies, such as those using data from high-risk cohorts like NAPLS^95^, AMP SCZ^97^, or KePROS^98^. Thirdly, specificity of our metrics to schizophrenia remains unclear, as network size and topography assessments are rare in functional connectivity research. In our previous FUNCATA study, DAN enlargement was minimal in bipolar disorder^37^, suggesting that this feature may reflect broader neuropathological severity or medication effects. Similarly, we found unilateral DMN enlargement in a case of depression with maladaptive daydreaming^38^, highlighting the importance of examining network metrics across disorders. Finally, we used k-means clustering solely on network size, as our goal was to determine whether a single, well-validated feature could reveal meaningful subgroups. Future work should assess whether incorporating additional features—such as TAI or other measures of topographic variability—produces similar or more granular subgroup patterns. Nonetheless, the observed TAI differences across these size-derived subgroups indicate a strong correspondence between network size and topographic abnormality.

## Conclusion

In summary, we replicated DAN enlargement in psychosis and additionally found DMN enlargement in this larger cohort, although LAN enlargement was not observed. Our findings support the potential of individualized estimates of network size and topography as biomarkers for risk prediction and treatment monitoring. Longitudinal studies will be essential to track changes in these measures over time, particularly in individuals at clinical high risk for psychosis, and to evaluate how they evolve with disease progression.

## Supplementary information

Supplemental Materials

## Data Availability

Data will be available upon request to the corresponding author.
