# Supplementary material for: Functional network comparative area and topography analysis (FUNCATA) in non-affective psychosis: a replication study
Source: Schizophrenia (Heidelb). 2026 Feb 17;12(1):32. doi: 10.1038/s41537-026-00736-z (PMC13022051; doi:10.1038/s41537-026-00736-z)
Supplement: Supplementary file 1 — Supplemental Materials [file 41537_2026_736_MOESM1_ESM.docx]

**SUPPLEMENTAL MATERIALS**

SUPPLEMENTAL FIGURES:

***
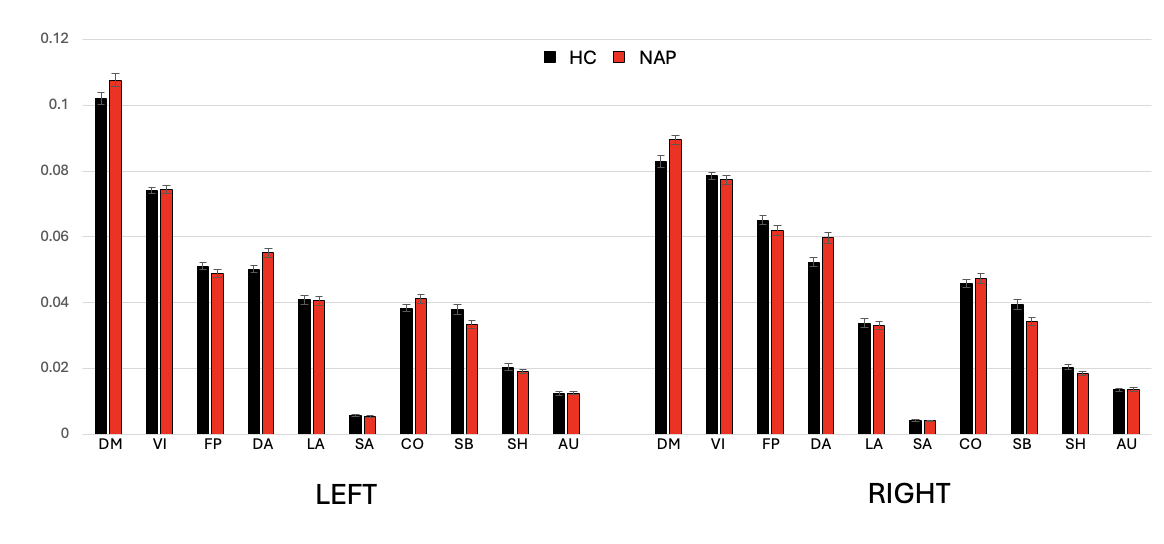
***

**Supplemental Figure 1. Size of functional connectivity networks in non-affective psychosis and healthy control participants in each hemisphere.** The figure compares the average relative network size of ten functional networks (relative to the total number of cortical surface vertices) in each hemisphere across non-affective psychosis (n=86; red bars) and healthy control (n=57; black bars) participants from the Human Connectome Project – Early Psychosis study. Error bars represent standard error. DM=default mode network. VI=visual network. FP=frontoparietal network. DA=dorsal attention network. LA=language network. SA=salience network. CO=cingulo-opercular network. SB=sensorimotor-body network. SH=sensorimotor-head network. AU=auditory network.


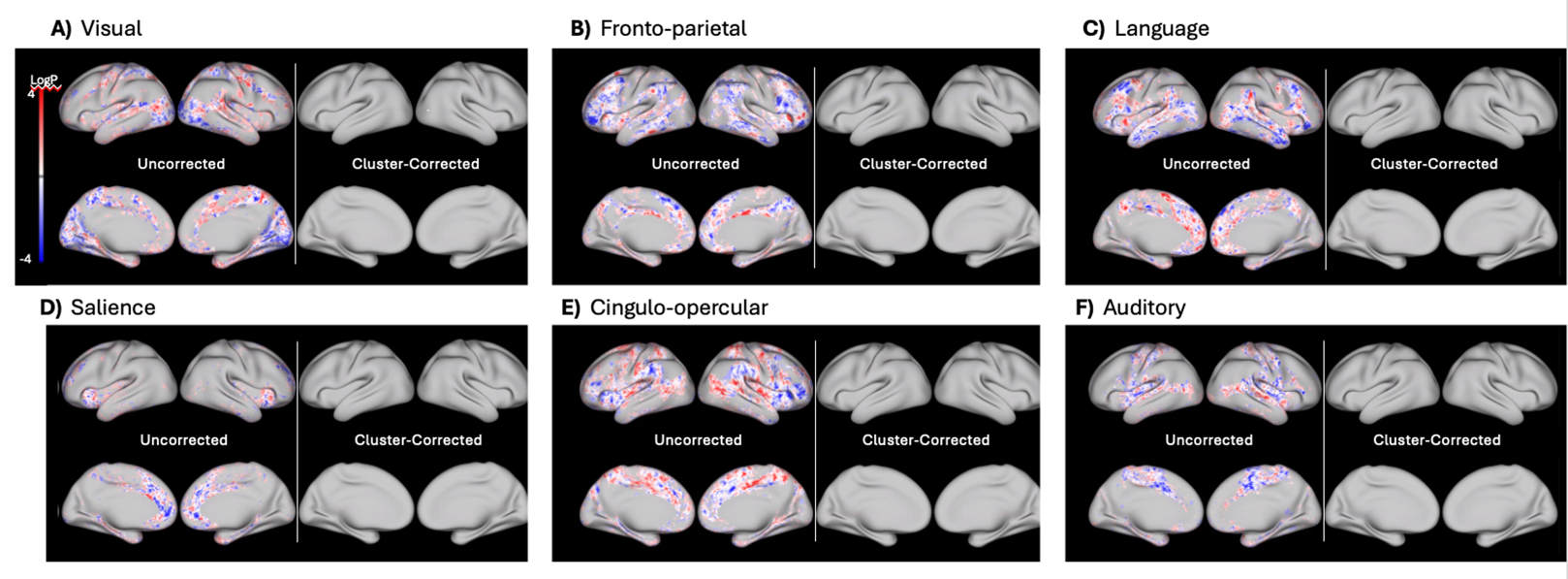


**Supplemental Figure 2. Group differences in functional network localization for networks showing no significant differences after cluster-correction.**  The figures are the results of chi-square tests at each surface vertex, comparing the proportion of individuals assigned to the network at that vertex in non-affective psychosis participants (n=86) to the proportion in healthy control participants (n=57). Color-coding is based on log_10_(p-values). Red shading indicates vertices where the network localizes more frequently in NAP participants. Blue shading indicates vertices where the network localizes more frequently in control participants. None of these 6 networks exhibited any significant clusters after permutation-based cluster-size correction.


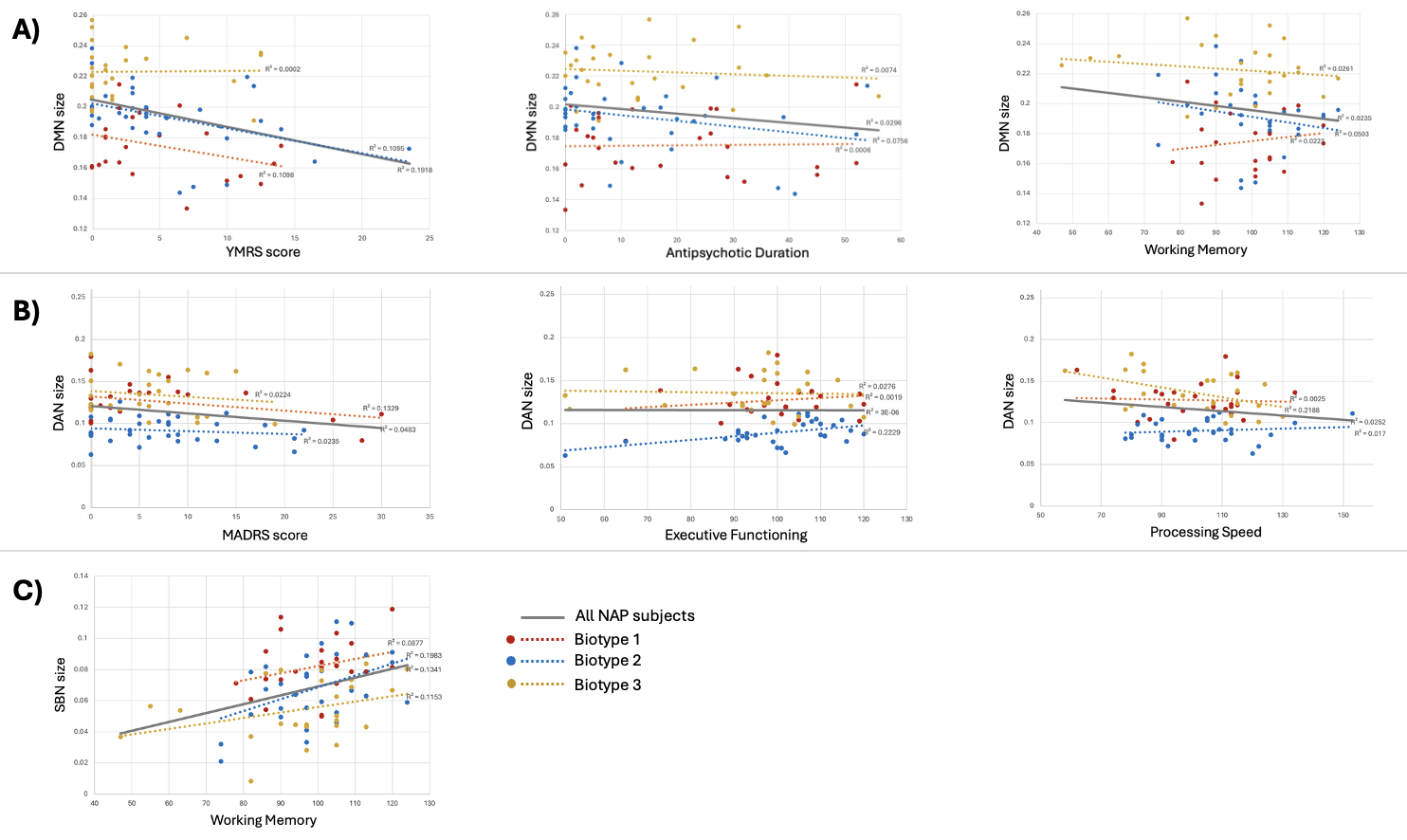
**Supplemental Figure 3. Functional network size relationships across all NAP subjects and within individual network size-derived biotypes.**  Panels illustrate clinically relevant associations for the default mode network (DMN; **A**), dorsal attention network (DAN; **B**), and sensorimotor-body network (SBN; **C**). Solid gray lines depict correlations across all individuals with non-affective psychosis (NAP), whereas colored dashed lines depict correlations within individual NAP biotypes. YMRS = Young Mania Rating Scale; MADRS = Montgomery–Åsberg Depression Rating Scale. Antipsychotic duration is reported in months. Neurocognitive scores were derived from the NIH Toolbox.

SUPPLEMENTAL TABLES:

**SUPPLEMENTAL TABLE 1. Functional network size in control and non-affective psychosis (NAP) groups**

Functional CON NAP F p Cohen’s D

Networks (%) (n=57) (n=86)

**Default Mode**

Total 0.185 (0.03) 0.197 (0.03) 6.31 **0.013*** 0.43

Left 0.102 (0.02) 0.108 (0.02) 3.65 0.058 0.33

Right 0.083 (0.02) 0.089 (0.01) 6.74 **0.010*** 0.44

**Visual**

Total 0.153 (0.02) 0.152 (0.02) 0.11 0.7 0.06

Left 0.074 (0.01) 0.074 (0.01) 0.01 0.9 0.02

Right 0.079 (0.01) 0.077 (0.01) 0.49 0.5 0.24

**Fronto-Parietal**

Total 0.116 (0.02) 0.111 (0.02) 2.48 0.1 0.27

Left 0.051 (0.01) 0.049 (0.01) 1.48 0.2 0.21

Right 0.065 (0.01) 0.062 (0.01) 2.05 0.2 0.24

**Dorsal Attention**

Total 0.103 (0.02) 0.115 (0.03) 7.99 **0.005**** 0.48

Left 0.050 (0.01) 0.055 (0.01) 5.88 **0.017*** 0.41

Right 0.052 (0.01) 0.060 (0.02) 9.05 **0.003**** 0.51

**Language**

Total 0.075 (0.02) 0.074 (0.02) 0.07 0.8 0.05

Left 0.041 (0.01) 0.041 (0.01) 0.02 0.9 0.02

Right 0.034 (0.01) 0.033 (0.01) 0.14 0.7 0.06

**Salience**

Total 0.010 (0.01) 0.010 (0.01) 0.27 0.6 0.09

Left 0.006 (0.00) 0.005 (0.00) 0.21 0.6 0.08

Right 0.004 (0.00) 0.004 (0.00) 0.26 0.6 0.09

**Cingulo-opercular**

Total 0.084 (0.02) 0.089 (0.03) 1.14 0.3 0.18

Left 0.038 (0.01) 0.041 (0.01) 1.88 0.2 0.23

Right 0.046 (0.01) 0.047 (0.01) 0.43 0.5 0.11

**Sensorimotor (Body)**

Total 0.077 (0.03) 0.068 (0.02) 5.32 **0.022*** 0.39

Left 0.038 (0.01) 0.033 (0.01) 4.99 **0.027*** 0.38

Right 0.039 (0.01) 0.034 ( 0.01) 5.35 **0.022*** 0.39

**Sensorimotor (Head)**

Total 0.041 (0.02) 0.038 (0.01) 1.99 0.2 0.24

Left 0.021 (0.01) 0.019 (0.01) 1.08 0.3 0.18

Right 0.021 (0.01) 0.019 (0.01) 2.89 0.09 0.29

**Auditory**

Total 0.026 (0.01) 0.026 (0.01) 0.01 0.9 0.02

Left 0.013 (0.00) 0.013 (0.00) 0.00 0.9 0.01

Right 0.014 (0.01) 0.014 (0.01) 0.02 0.9 0.03

* p<0.05. **p<0.005

**SUPPLEMENTAL TABLE 2. Functional network size in control (CON) and non-affective psychosis (NAP) groups using a restricted NAP subgroup with equal sample size and reduced age disparity relative to controls (sensitivity analysis).**

Functional CON NAP F p

Networks (%) (n=57) (n=57)

**Default Mode** 0.185 (0.03) 0.197 (0.03) 4.75 **0.031***

**Visual** 0.153 (0.02) 0.154 (0.02) 0.17 0.7

**Fronto-Parietal** 0.116 (0.02) 0.111 (0.02) 2.13 0.15

**Dorsal Attention** 0.103 (0.02) 0.113 (0.03) 4.96 **0.028***

**Language** 0.075 (0.02) 0.076 (0.02) 0.03 0.9

**Salience** 0.010 (0.01) 0.009 (0.01) 0.90 0.3

**Cingulo-opercular** 0.084 (0.02) 0.090 (0.03) 1.40 0.2

**Sensorimotor (Body)** 0.077 (0.03) 0.067 (0.02) 4.68 **0.033***

**Sensorimotor (Head)** 0.041 (0.02) 0.038 (0.01) 1.22 0.3

**Auditory** 0.026 (0.01) 0.026 (0.01) 0.03 0.9

To reduce baseline age differences between groups, the youngest non-affective psychosis (NAP) participants were excluded, resulting in equal group sizes (n = 57 per group) and a substantially reduced age difference relative to the full sample. In the restricted sample, mean age (± s.d.) was 24.3 ± 4.2 years in healthy controls and 23.3 ± 2.9 years in the NAP group. * p<0.05.

**SUPPLEMENTAL TABLE 3. Functional network size in control and non-affective psychosis (NAP) groups using a restricted NAP subgroup with equal sample size and reduced framewise displacement (FD) relative to controls (sensitivity analysis).**

Functional CON NAP F p

Networks (%) (n=57) (n=57)

**Default Mode** 0.185 (0.03) 0.197 (0.03) 5.42 **0.022***

**Visual** 0.153 (0.02) 0.151 (0.02) 0.19 0.7

**Fronto-Parietal** 0.116 (0.02) 0.110 (0.02) 3.22 0.08

**Dorsal Attention** 0.103 (0.02) 0.116 (0.03) 8.43 **0.004****

**Language** 0.075 (0.02) 0.076 (0.02) 0.02 0.9

**Salience** 0.010 (0.01) 0.010 (0.01) 0.13 0.7

**Cingulo-opercular** 0.084 (0.02) 0.090 (0.03) 0.05 0.8

**Sensorimotor (Body)** 0.077 (0.03) 0.067 (0.02) 5.76 **0.018***

**Sensorimotor (Head)** 0.041 (0.02) 0.038 (0.01) 2.50 0.1

**Auditory** 0.026 (0.01) 0.026 (0.01) 0.27 0.6

To reduce between-group differences in head motion, the NAP sample was restricted by excluding participants with the highest framewise displacement, yielding equal group sizes (n = 57 per group). Mean FD (± s.d.) in the restricted sample was 0.117 ± 0.04 in controls and 0.115 ± 0.03 in the NAP group, compared with 0.117 ± 0.04 and 0.160 ± 0.09, respectively, in the full sample. * p<0.05. **p<0.005**.**

**SUPPLEMENTAL TABLE 4. Topographic Abnormality Index (TAI) in control and non-affective psychosis (NAP) groups.**

Functional CON NAP F p Cohen’s D

Networks (%) (n=57) (n=86)

**Default Mode** 0.025 (0.02) 0.036 (0.02) 11.57 **0.00088**** 0.59

**Visual** 0.029 (0.03) 0.039 (0.04) 2.46 0.1 0.27

**Fronto-Parietal** 0.042 (0.02) 0.054 (0.03) 5.69 **0.018***  0.41

**Dorsal Attention** 0.047 (0.03) 0.070 (0.04) 12.87 **0.00047***** 0.62

**Language** 0.070 (0.03) 0.085 (0.05) 4.32 **0.04***  0.36

**Salience** 0.323 (0.13) 0.361 (0.14) 2.51 0.1 0.27

**Cingulo-opercular** 0.058 (0.03) 0.081 (0.04) 13.89 **0.00028***** 0.65

**Sensorimotor (Body)** 0.037 (0.03) 0.036 (0.03) 0.01 0.9 -0.01

**Sensorimotor (Head)** 0.068 (0.07) 0.063 (0.05) 0.24 0.6 -0.09

**Auditory** 0.136 (0.08) 0.156 (0.08) 1.89 0.2 0.24

* p<0.05. **p<0.005. ***p<0.0005

**SUPPLEMENTAL TABLE 5. Topographic Abnormality Index (TAI) in control (CON) and non-affective psychosis (NAP) groups using a restricted NAP subgroup with equal sample size and reduced age disparity relative to controls (sensitivity analysis).**

Functional CON NAP F p

Networks (%) (n=57) (n=57)

**Default Mode** 0.025 (0.02) 0.038 (0.02) 15.23 **0.00016*****

**Visual** 0.029 (0.03) 0.044 (0.04) 4.62 **0.034***

**Fronto-Parietal** 0.042 (0.03) 0.054 (0.03) 6.80 **0.01***

**Dorsal Attention** 0.047 (0.03) 0.073 (0.04) 14.29 **0.00025*****

**Language** 0.070 (0.03) 0.092 (0.05) 8.49 **0.0043****

**Salience** 0.323 (0.14) 0.378 (0.14) 4.54 **0.035***

**Cingulo-opercular** 0.058 (0.03) 0.089 (0.04) 23.03 **0.000005******

**Sensorimotor (Body)** 0.037 (0.03) 0.040 (0.04) 0.39 0.5

**Sensorimotor (Head)** 0.068 (0.07) 0.065 (0.05) 0.04 0.8

**Auditory** 0.136 (0.08) 0.161 (0.09) 2.46 0.1

To reduce baseline age differences between groups, the youngest non-affective psychosis (NAP) participants were excluded, resulting in equal group sizes (n = 57 per group) and a substantially reduced age difference relative to the full sample. In the restricted sample, mean age (± s.d.) was 24.3 ± 4.2 years in healthy controls and 23.3 ± 2.9 years in the NAP group. * p<0.05. **p<0.005. ***p<0.0005. ****p<0.00005.

**SUPPLEMENTAL TABLE 6. Topographic Abnormality Index (TAI) in control and non-affective psychosis groups using a restricted NAP subgroup with equal sample size and reduced framewise displacement (FD) relative to controls (sensitivity analysis).**

Functional CON NAP F p

Networks (%) (n=57) (n=57)

**Default Mode** 0.025 (0.02) 0.034 (0.02) 7.23 **0.0084***

**Visual** 0.029 (0.03) 0.037 (0.04) 1.28 0.26

**Fronto-Parietal** 0.042 (0.03) 0.047 (0.02) 1.43 0.2

**Dorsal Attention** 0.047 (0.03) 0.064 (0.04) 7.19 **0.0085***

**Language** 0.070 (0.03) 0.079 (0.04) 1.74 0.19

**Salience** 0.323 (0.14) 0.331 (0.13) 0.11 0.7

**Cingulo-opercular** 0.058 (0.03) 0.075 (0.04) 6.69 **0.01***

**Sensorimotor (Body)** 0.037 (0.03) 0.033(0.03) 0.28 0.6

**Sensorimotor (Head)** 0.068 (0.07) 0.052 (0.05) 2.03 0.2

**Auditory** 0.136 (0.08) 0.155 (0.08) 1.52 0.2

To reduce between-group differences in head motion, the NAP sample was restricted by excluding participants with the highest framewise displacement, yielding equal group sizes (n = 57 per group). Mean FD (± s.d.) in the restricted sample was 0.117 ± 0.04 in controls and 0.115 ± 0.03 in the NAP group, compared with 0.117 ± 0.04 and 0.160 ± 0.09, respectively, in the full sample. * p<0.05.

**SUPPLEMENTAL TABLE 7. Demographic and clinical characteristics of non-affective psychosis (NAP) biotypes**

Characteristic Biotype 1 Biotype 2 Biotype 3

(n=25) (n=35) (n=26)

**Age (s.d.)** 21.0 (3.0) 22.4 (3.6) 21.4 (3.2)

**Sex (%)**

Female 5 (20.0) 14 (40.0) 8 (30.8)

Male 20 (80.0) 21 (60.0) 18 (69.2)

**Ethnicity (%)**

Asian 1 (4.0) 3 (8.6) 1 (3.8)

Black 14 (56.0) 15 (42.9) 12 (46.2)

White 10 (40.0) 13 (37.1) 13 (50.0)

Mixed 0 1 (2.9) 0

Other* 0 3 (8.6) 0
